# Mastering Proton Activities in Aqueous Batteries

**DOI:** 10.1002/adma.202407852

**Published:** 2024-09-03

**Authors:** Leiting Zhang, Chao Zhang, Erik J. Berg

**Affiliations:** ^1^ Department of Chemistry–Ångström Laboratory Uppsala University Box 538 Uppsala 751 21 Sweden

**Keywords:** aqueous batteries, proton activities, reaction kinetics, solid‐electrolyte interphase, thermodynamics, water electrolysis

## Abstract

Advanced aqueous batteries are promising solutions for grid energy storage. Compared with their organic counterparts, water‐based electrolytes enable fast transport kinetics, high safety, low cost, and enhanced environmental sustainability. However, the presence of protons in the electrolyte, generated by the spontaneous ionization of water, may compete with the main charge‐storage mechanism, trigger unwanted side reactions, and accelerate the deterioration of the cell performance. Therefore, it is of pivotal importance to understand and master the proton activities in aqueous batteries. This Perspective comments on the following scientific questions: Why are proton activities relevant? What are proton activities? What do we know about proton activities in aqueous batteries? How do we better understand, control, and utilize proton activities?

## Graphical abstract

Advanced aqueous batteries are promising alternatives for grid energy storage. Understanding and mastering the proton activities in aqueous electrolytes will guide the design of sustainable and long‐lasting aqueous batteries. This Perspective comments on the current understanding of the thermodynamic and kinetic perspectives of proton activities and highlights future research directions for aqueous batteries.

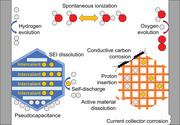

## 1 Why are Proton Activities Relevant to Aqueous Batteries?

Benefitting from the unique properties of water as an electrolyte solvent, aqueous batteries are a sustainable alternative solution for large‐scale energy storage. The low viscosity of water provides fast reaction and transport kinetics and its high dielectric constant makes most salts soluble. The amphoteric nature of water to spontaneously ionize provides aqueous electrolytes with inseparable agents for charge transport and storage, namely the proton (H^+^), hydronium (H_3_O^+^), and/or hydroxide (OH^−^) ions. Compared to all other electrolyte ions, the proton has the smallest radius and lowest mass, principally providing aqueous batteries with the highest power/capacity capability possible. In aqueous solutions, proton transport generally follows two mechanisms, namely the Grotthuss and the vehicular mechanisms. In the former, protons are hopping between neighboring water molecules through the hydrogen bond network, which enables ultrafast proton conduction. In contrast, the vehicular mechanism involves the physical movement of hydronium ions through the electrolyte, which is much sluggish compared to the former.^[^ ^1^ ^]^

**Figure 1:**
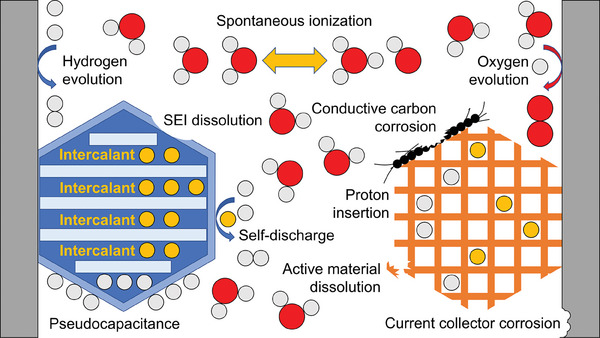
A summary of proton‐participating reactions taking place in aqueous cells.

On the flip side, the fast kinetics and high solubilizing power also promote side reactions and cause accelerated battery failure. For example, hydrogen evolution reaction (HER) may take place on the anode, competing against desired charge‐storage reactions and posing potential safety risks. Protons can promote side reactions that generate byproducts, such as metal oxides or hydroxides, which may precipitate on the electrode surface and compromise electrode ion transport properties. Moreover, acidic electrolytes may accelerate the corrosion of electrode materials and cell components, reducing the shelf life and calendar life of aqueous batteries. Common chemical and electrochemical reactions in batteries involving protons may be grouped into four categories, as illustrated in **Figure** **1**, which will be further elaborated in Section 3.

Historically, proton‐coupled electron transfer processes have been extensively studied in chemistry and biochemistry,^[^ ^2^, ^3^ ^]^ where the transfer of an electron is directly linked to the transfer of a proton. These reactions also play a decisive role in the charge transfer in aqueous batteries, such as lead‐acid batteries (PbO_2_ + 4H^+^ + SO_4_^2 −^ + 2*e* ^−^⇌PbSO_4_ + 2H_2_O) and Ni‐based alkaline batteries (NiOOH + H_2_O + *e* ^−^⇌Ni(OH)_2_ + OH^−^). The type of cell chemistry is closely related to the surface redox reactions found in pseudocapacitors^[^ ^4^ ^]^ and has been revived recently under the name of proton batteries.^[^ ^5^ ^]^ On the other hand, exploring aqueous batteries with mono‐ and multi‐valent charge carriers (e.g., Li^+^, Na^+^, K^+^, Zn^2+^, Al^3+^, etc.) is also of high interest, where the involvement of the proton insertion is controversial. In both cases, one would need to understand, control, and utilize proton activities in aqueous batteries, which is essential to designing next‐generation sustainable and high‐performance aqueous batteries for grid energy storage. This calls for joint efforts from experiments and molecular modeling^[^ ^6^ ^]^ and motivates us to write this Perspective and offer an outlook on future research opportunities.

In the following, we will first rigorously define proton activities, which serve as a common ground for both experimental and theoretical investigations. Then, we will discuss examples of aqueous batteries, where proton activities play a key role. Finally, we will list a number of important bullet points for future studies.

## 2 What are Proton Activities?

In order to set the stage, it is necessary to first introduce the fundamental thermodynamic quantities related to protons in aqueous electrochemistry. The first key quantity is the electrochemical potential of protons ${\tilde{\mu }}_{H^{+}}$ in an electrolyte solution, which includes contributions from both the chemical potential $\mu_{H^{+}},$ and the Galvani potential ϕ as

(1) $${\tilde{\mu }}_{H^{+}}=\;\mu_{H^{+}}+F\phi$$

where *F* is the Faraday constant. For the sake of simplicity, we omitted the symbol of the phase to which the protons belong, which will appear in parenthesis when needed.

The chemical potential of protons is related to the activity of protons $a_{H^{+}}$ in the following way:

(2) $$\mu_{H^{+}}=\;{\mu^{\circ }}_{H^{+}}+RT\ln a_{H^{+}}$$

where *R* is the universal gas constant and *T* is the temperature. The activity is connected to the concentration of proton via $a_{H^{+}}=\;\gamma_{H^{+}}\cdot c_{H^{+}}/{c^{\circ }}_{H^{+}}$ in which $\gamma_{H^{+}}$ is the so‐called activity coefficient and ${c^{\circ }}_{H^{+}}$ is the standard concentration of 1 molar (mol per liter). The second term on the right‐hand side of Equation (2) can be expressed using the pH scale as − 2.303*RT*pH, i.e., $a_{H^{+}}=10^{-\mathrm{pH}}$. It is worth noting that the activity of protons $a_{H^{+}}$ is a narrower concept while the proton (re)activity in chemical equilibrium is determined by ${\tilde{\mu }}_{H^{+}}$ instead.

The Galvani potential ϕ in Equation (1) contains two terms as well: the Volta potential ψ and the surface potential χ. The Volta potential is usually assumed to be zero and the surface potential that manifests itself at the liquid‐vapor interface is about a few hundred millivolts. It may be odd at first glance that the liquid‐vapor interface needs to be brought up here to define the electrochemical potential of protons, which will become clear later. At this stage, what should be noted is there is no way to separate the contribution from the chemical potential and the Galvani potential without introducing further assumptions.^[^ ^7^ ^]^ Nevertheless, Galvani potential is a useful mathematical construct to build up the thermodynamic framework in electrochemistry. A similar and familiar example of this kind is the wavefunction used in quantum mechanics, which is also a non‐measurable but useful quantity.

Once we have defined the electrochemical potential of protons, the immediately related quantity is the proton work function $W_{H^{+}}$. The work function of protons stands for the work that needs to be carried out to bring a proton from an aqueous solution at the standard conditions to the immediate outside of a liquid‐vapor interface. It is just the opposite of the ${\tilde{\mu }}_{H^{+}}$:

(3) $$W_{H^{+}}=\;-{\tilde{\mu }}_{H^{+}}$$

From this expression, it is now clear that the electrochemical potential of protons includes a contribution of a liquid‐vapor interface implicitly and depends significantly on the nature of solvation. That is the reason why the surface potential shows up in the Galvani potential. More importantly, the proton work function is an experimentally measurable quantity, which is ≈11.36 eV from the seminal work of Fawcett using the Kenrick cell.^[^ ^8^ ^]^ This provides a way to determine the standard free energy for the hydrogen evolution reaction, hence the standard hydrogen electrode (SHE) scale.

**Figure 2:**
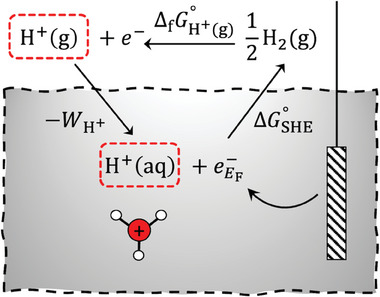
A thermodynamic cycle for defining the standard hydrogen electrode (SHE).

As shown in **Figure** **2**, the standard free energy change for the standard hydrogen electrode $\Delta G_{H^{+}}^{\circ }$ can be computed from a thermodynamic cycle. Given that the standard formation energy of H^+^ in the gas phase $\Delta_{f}G_{H^{+(g)}}^{\circ }$ is 15.81 eV and the work function of protons is 11.36 eV, this leads to $\Delta G_{H^{+}}^{\circ }$ of 4.45 eV.^[^ ^9^ ^]^ Therefore, the conversion between the absolute vacuum scale and the SHE scale is $E_{X^{-}/X}^{\mathrm{SHE}}=\;\left(\Delta G_{X^{-}/X}^{\circ }-\;\Delta G_{H^{+}}^{\circ }\right)/F$.^[^ ^10^ ^]^ That is why $E_{\mathrm{HER}}^{\mathrm{SHE}}=\;0$ by definition under standard conditions. Furthermore, it shows that redox levels are thermodynamic levels rather than electronic levels, which differ from each other by the reorganization energy.^[^ ^11^ ^]^

In addition to the redox levels, p*K*_a_ reflects the proton activity under the open circuit conditions. For a species like HX, it is linked to the equilibration constant *K*_*a*, HX_ for the corresponding proton dissociation reaction in aqueous solutions (in contrast to proton affinity which is a gas phase quantity).

(4) $$pK_{a,\;\mathrm{HX}}=\;-\;\log\left(K_{a,\;\mathrm{HX}}\right)=\;-\log\left(\frac{\left[H^{+}\right]\left[X^{-}\right]}{\left[\mathrm{HX}\right]}\right)$$

where [⋅⋅⋅] indicates the concentration of the corresponding species normalized by the standard concentration.

For metal surfaces at high potential and (semi)conducting metal oxide surfaces, there are plenty of surface sites that can receive or donate protons. This leads to the definition of surface p*K*_a_ for species like MOH_2_^+^ (aq) and MOH (aq), where M is the metal ion at solid‐liquid interfaces and the formation of the electric double layers due to the proton charge.^[^ ^12^ ^]^

## 3 What do We Know about Proton Activities in Aqueous Batteries?

The nature and extent of proton involvement in charge storage or other side processes is largely determined by its activity, which in turn is related to the concentration and environment of the protons in the electrolyte, as discussed above. The most immediate chemical environment is normally the surrounding solvation shell. Water or other solvating molecules bond to the proton and compete to electrically shield it from the electrostatic field primarily induced by neighboring ions and/or the electrode surface. Solvent‐shielding may break down at higher ion concentrations or in the vicinity of the electrode surface as the protons then more strongly interact with neighboring charges. Although proton activity is a strictly thermodynamic notion, its influence on electrochemical processes is often difficult to separate from kinetic factors. Even the reversible electrode potentials for reactions involving protons are for the same reason difficult to determine. Despite the complexity, understanding the electrochemical mechanisms of protons in various aqueous electrolytes is essential for designing and optimizing sustainable energy storage devices. Based on the nature of the proton‐involving (electro)chemical reactions, we categorize representative scenarios into 1) bulk and surface proton storage, 2) spontaneous ionization/dissociation of electrolyte and electrode species, 3) electrochemical decomposition of cell components, and 4) self‐discharge/dissolution. In the following, we elaborate on each point with our perspectives, complemented by examples from recent publications. A table summarizing all factors is presented at the end of this section (**Table** **1**).

### 3.1 Bulk and Surface Proton Storage

In aqueous electrochemical energy storage systems, charge‐storage processes can be divided into faradaic and non‐faradaic reactions, which describe how energy is stored and released at the electrode‐electrolyte interface. Faradaic reactions in classic battery materials involve the transfer of charge through redox processes, while non‐faradaic reactions taking place in conventional capacitors and electrochemical double‐layer capacitors are built upon electrostatic charge separation. A unique category of systems possessing fast and reversible redox reactions at the electrode surface and subsurface regions are known as pseudocapacitors. In this subsection, faradaic‐type proton storage reactions will be briefly discussed.

**Figure 3:**
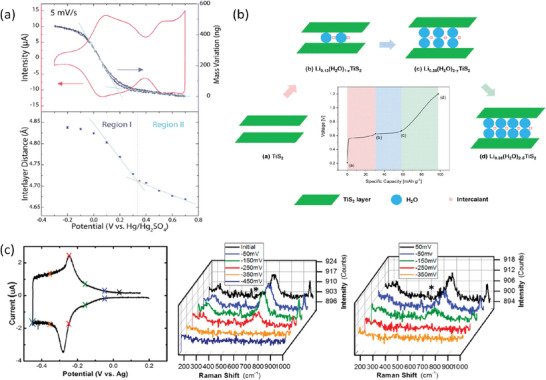
a) EQCM measurement and the corresponding interlayer distance as functions of the applied potential on composite electrodes of H_4_IrO_4_. Reproduced with permission.^[^ ^17^ ^]^ Copyright 2022, American Chemical Society. b) Reversible hydration of TiS_2_ in aqueous batteries. Reproduced under terms of the CC‐BY license.^[^ ^24^ ^]^ Copyright 2024, L. Zhang et al., published by the American Chemical Society. c) In situ Raman spectroscopy (left) of WO_3_∙2H_2_O during the cathodic (middle) and anodic (right) scans. Adapted with permission.^[^ ^25^ ^]^ Copyright 2017, American Chemical Society.

The presence of multiple charge carriers in the aqueous electrolyte leads to competition among proton, hydronium, and eventual metal cations to react with the electrode.^[^ ^13^, ^14^, ^15^, ^16^ ^]^ For instance, Lemaire et al. studied the proton storage mechanism in H*_x_* IrO_4_ by combining electrochemical quartz‐crystal microbalance (EQCM) and X‐ray diffraction (XRD).^[^ ^17^ ^]^ The former technique demonstrated the preferential intercalation of hydronium ions at lower potentials and de‐solvated protons at higher (**Figure** **3**a). The potential‐dependent intercalation of first H_3_O^+^ and then H^+^ was confirmed by monitoring the changes in interlayer distance between the IrO_4_ slabs during cycling. Solid‐state nuclear magnetic resonance (ssNMR) spectroscopy is another powerful technique to probe local environments around the nuclei. For example, Guo et al. assigned the broad signal at 8.3 ppm chemical shift of the ^1^H NMR spectrum to water molecules confined at the surface of α‐MoO_3_, as compared to the 4.7 ppm signal for bulk crystal water.^[^ ^18^ ^]^ Paik et al. conducted ^2^H NMR experiments to investigate the interaction of deuteron with highly defective paramagnetic materials, such as electrolytic manganese dioxide.^[^ ^19^ ^]^ They were able to assign deuterons at different positions (at cation vacancy sites, or in the so‐called 1 × 1 and 1 × 2 tunnels). Other studies have shown that the desolvation and insertion mechanisms of protons may be affected by the local structure of the electrode material. Geng et al. reported a crystalline‐surface‐dependent dehydration process, allowing bare protons to be inserted through the anatase TiO_2_ (001) surface but not the (101) surface in an acidic electrolyte (0.5 m of H_2_SO_4_).^[^ ^20^ ^]^ Meanwhile, Makivić et al. observed bulk proton insertion in nanostructured TiO_2_ electrodes composed of either anatase or amorphous TiO_2,_ using a buffered mild acidic electrolyte (1 M of acetate buffer at pH = 5).^[^ ^21^ ^]^ Not only may there be a competition between de‐/solvated protons, but also between protons and metal ions. For example, MnO_2_ was traditionally believed to undergo direct Zn‐insertion on discharge in aqueous Zn‐ion batteries. However, Sun et al. suggested a sequential H^+^/Zn^2+^ co‐insertion mechanism, in which the first half of the discharge capacity originated from proton insertion from the electrolyte while ZnMn_2_O_4_ was identified as the end‐discharge product by *ex situ* XRD.^[^ ^14^ ^]^ In contrast, Yuan et al. conducted advanced electron microscopy and concluded that Zn^2+^ insertion was unlikely in the aqueous system and the discharge products should be H*_x_* MnO_2_ (MnO_2_ + *xe* ^−^ + *x*H^+^⇌H_*x*_MnO_2_) and zinc hydroxide sulfate.^[^ ^22^ ^]^ The competing intercalation between proton and other cations (Na^+^, Zn^2+^, Mg^2+^, and Al^3+^) into layered V_2_O_5_ was studied by Lee et al. using beaker cells with a large amount of electrolyte. By coupling several X‐ray techniques with electron microscopy, it was found that proton insertion could proceed before the insertion of other cations in low pH conditions (pH ≤ 3).^[^ ^23^ ^]^ Recently, we proposed a reversible hydration model to rationalize the peculiar multi‐plateaued voltage curve of TiS_2_ in dilute aqueous electrolytes. By performing synchrotron‐based *operando* XRD and molecular dynamics (MD) simulation, co‐intercalation of hydrated intercalants (Li(H_2_O)_*x*_^+^ and/or H_3_O^+^ ions) in TiS_2_ was confirmed (Figure 3b).^[^ ^24^ ^]^ All these examples highlight the complex scenarios when multiple charge carriers present in the electrolyte are competing for charge compensation in aqueous batteries and the necessity of implementing advanced characterization techniques to depict the underlying mechanism.

Apart from the typical insertion materials (such as certain metal oxides, Prussian blue analogs, 2D carbides/nitrides), protons can also be stored through surface redox reactions. For example, hydrated RuO_2_ is a classic pseudocapacitive material that undergoes surface redox reactions, thanks to the fast proton transfer through the hydrous boundaries of RuO_2_ (RuO_*x*_(OH)_*z*_ + δ*e* ^−^ + δH^+^⇌RuO_*x* − δ_(OH)_*z* + δ_).^[^ ^26^, ^27^ ^]^ The magnitude of the capacitance in acidic electrolytes can be tuned by optimizing the water content in the hydrous RuO_2_∙*n*H_2_O sample.^[^ ^28^, ^29^ ^]^ Similarly, the presence of structural water may transform battery‐like proton storage in anhydrous WO_3_ into an ideally pseudocapacitive behavior in WO_3_∙2H_2_O. Mitchell et al. performed in situ Raman spectroscopy of hydrous WO_3_∙2H_2_O samples during electrochemical cycling (Figure 3c) and observed that the local structure was preserved after cycling without loss of interlayer and covalently bound water.^[^ ^25^ ^]^ Interestingly, there was a complete loss of Raman peaks at potentials lower than −0.25 V, after which the CV showed a predominantly capacitive feature. The protonation process was accompanied by the concomitant reduction of W^6+^ to W^5+^, which triggered the semiconductor‐to‐metal transition of the hydrous WO_3_∙2H_2_O phase, resulting in the extinction of the Raman signal. Meanwhile, the contribution between diffusion‐limited and capacitive processes can be differentiated based on the following relationship:

(5) $$i\;\left(V\right)=k_{1}\;v+k_{2}v^{1/2}$$

where i is the current at a particular potential, *v* is the sweep rate, and k_1_ and k_2_ are rate coefficients. It was found that the majority of the current (87%) in WO_3_∙2H_2_O was from a capacitive process, while the same process only contributed to 39% of the current in WO_3_. This study highlights the unique pseudocapacitive proton storage mechanism and the critical role of water in facilitating fast proton conduction in hydrated metal oxides.

In addition, organic materials can also store protons through the reversible conversion of carbonyl (─C═O) or imino (═N─) redox centers (─C═O +*e* ^–^ + H^+^ ⇌ ─C─O─H). For instance, an all‐quinone symmetric aqueous battery was constructed by assembling 1,2‐dihydroxyanthraquinone (alizarin) as both the cathode and the anode.^[^ ^30^ ^]^ Proton‐coupled electron transfer takes place by alizarin either accepting or donating two electrons and two protons through the para‐quinone and the ortho‐hydroquinone motifs. Since this Perspective focuses mainly on inorganic electrode materials, readers are advised to refer to other review papers on this topic.^[^ ^31^, ^32^, ^33^ ^]^

### 3.2 Spontaneous Ionization/Dissociation

**Figure 4:**
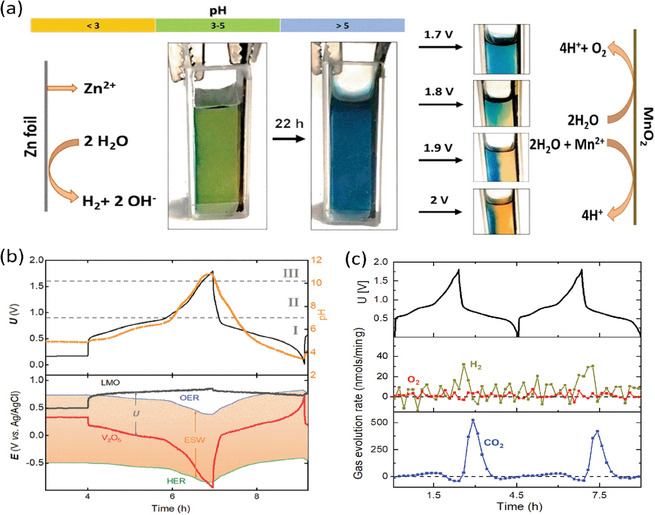
a) Visualization of the dynamic pH change of an aqueous electrolyte. Reproduced with permission.^[^ ^34^ ^]^ Copyright 2022, Elsevier. b) dynamic electrolyte ESW, reconstructed by monitoring the electrode potential and adjacent pH simultaneously, and c) *operando* gas analysis of V_2_O_5_ || LiMn_2_O_4_ aqueous full‐cells. Reproduced with permission.^[^ ^35^ ^]^ Copyright 2023, Wiley.

Although the proton activity $a_{H^{+}}$ is an intensive property, it is neither constant nor the same throughout the electrolyte during battery cycling. Experimentally, one may qualitatively assess the electrolyte acidity using pH indicators. For example, Perez‐Antolin et al. visualized the dynamic change of an aqueous electrolyte containing bromocresol green for aqueous Zn‐ion batteries (**Figure** **4**a).^[^ ^34^ ^]^ The spontaneous corrosion on the Zn anode released H_2_ and turned the local electrolyte more alkaline, while the electrolytic MnO_2_ deposition reaction on the cathode generated new protons and turned the local electrolyte more acidic. The dynamic tracking of the electrolyte acidity has also been done by incorporating a pH electrode into the cell configuration. Xu et al. showed that the apparent pH increased at the negative electrode (Figure 4b) on charge because of hydrogen evolution and proton storage reactions, and vice versa on discharge.^[^ ^35^ ^]^ Importantly, as has been pointed out, pH is only strictly defined in dilute ion solutions and proton activity is difficult to determine precisely in electrolytes of higher ion concentrations or the vicinity of the electrode surface.^[^ ^36^ ^]^ Nevertheless, $a_{H^{+}}$ is generally higher for electrolytes of higher ionic strength, be it due to the addition of monovalent cations^[^ ^36^ ^]^ or multivalent metal ion complexes of the type [M(H_2_O)_6_]^n+^ (M = Al, Fe, Zn, Mn, etc.).^[^ ^37^, ^38^ ^]^ For instance, electrolyte acidifies as a result of transition metal dissolution from electrode materials, especially in dilute aqueous electrolytes. A typical case is vanadium pentoxide (V_2_O_5_), which forms a series of polyvanadates (VO^2+^, HV_10_O_28_^5−^, V_4_O_12_^4−^, VO_4_^3−^, etc.) upon dissolution along with the release of protons.^[^ ^35^ ^]^ Not only the active materials may dissolve, but also the electrode current collectors. Aluminum applied in modern Li‐ion batteries is not suitable as current collectors for aqueous batteries because of its low electrode potential (−1.68 V vs SHE) and the dissolution of its surface passivation layer in acidic environments (Al_2_O_3_ + 6H^+^ → 2Al^3 +^ + 3H_2_O).^[^ ^39^ ^]^ Therefore, it is highly advised to review the corresponding Pourbaix diagram and understand the kinetic aspects of every electrode component of the battery.

### 3.3 Electrochemical Decomposition

For aqueous batteries, proton activity also largely determines the electrochemical stability window (ESW) of the electrolyte. Hydrogen evolution is the most challenging side reaction (2H^+^ + 2*e* ^−^ → H_2_) and its electrode potential *E_HER_* limits the cathodic ESW according to the Nernst equation. When the negative electrode is operated below *E_HER_*, protons are consumed, H_2_ evolves, and the local $a_{H^{+}}$ drops as OH^−^ forms. Consequently, HER is partially self‐inhibited as *E_HER_* shifts negatively when the electrolyte turns more alkaline (as demonstrated in Figure 4b). However, the negative electrode potential eventually crosses *E_HER_* and H_2_ starts to evolve (Figure 4c). At the positive electrode, $a_{H^{+}}$ rather increases and the electrolyte acidifies as a result of water oxidation (2H_2_O → 4*e* ^−^ + 4H^+^ + O_2_). Oxygen evolution reaction (OER) is, however, sluggish and O_2_ is typically not observed despite the positive electrode operating beyond the *E_OER_*.^[^ ^35^ ^]^ Instead, the conductive carbon is observed to readily corrode (C + 2H_2_O → 4*e* ^−^ + 4H^+^ + CO_2_) and evolve CO_2_ (Figure 4c), which may subsequently cross over to the negative electrode, where it is chemically consumed in the alkaline environment ($CO_{2}+2OH^{-}\to {\mathrm{CO}}_{3}^{2-}+H_{2}O$).

In the context of highly concentrated electrolytes, otherwise called water‐in‐salt electrolytes (WiSEs), it is equally critical to establish a fundamental understanding of the thermodynamic and kinetic contributions to parasitic water electrolysis. Dubouis et al. observed that *E_OER_* increased with the electrolyte salt concentration, but there was no clear correlation between *E_HER_* and electrolyte salt concentration.^[^ ^40^ ^]^ More recently, Zhao et al. proposed that the “seemingly unchanged” *E_HER_* could result from 1) a positive shift from the liquid junction potential and increased proton activity, and 2) a negative shift from the sluggish HER kinetics in highly concentrated electrolytes. The authors also found that impurities in the commercial LiTFSI salt (98%) could significantly suppress the HER kinetics in WiSEs, compared to LiTFSI with a higher purity of 99.95%.^[^ ^41^ ^]^

### 3.4 Self‐Discharge/Dissolution

Although the gradient in $a_{H^{+}}$ established through HER and OER kinetically expands the corresponding aqueous electrolyte ESW beyond 1.23 V, it is only maintained as long as H_2_O splits. The same side reactions cause the cell to self‐discharge as long as any of the electrodes reside outside the ESW after the charging step ends (e.g., 2Li_*x*_(Host) + 2*x*H_2_O → 2Host + 2*x*LiOH + H_2_).^[^ ^15^, ^42^ ^]^ The kinetic broadening of ESW and minimization in self‐discharge are most successfully realized in concentrated aqueous electrolytes, whereby a strong gradient in $a_{H^{+}}$ can be maintained owing to the restrained mobility of protons. For instance, Li et al. reported that the water network was disrupted by the presence of other cations (e.g., Li^+^, K^+^, Cs^+^, and TBA^+^) in the aqueous electrolyte. Fourier‐transformed infrared spectroscopy (FTIR) analysis and ab initio molecular dynamics (AIMD) simulation confirmed that protons were confined in the hydration shell of K^+^, which could effectively suppress the HER current by more than ten times.^[^ ^43^ ^]^ This agrees well with a previous MD study with machine learning potential (MDP), which demonstrated a strong effect of salt concentration on the reduction of proton mobility.^[^ ^44^ ^]^ It provides new insights into designing new electrolytes with regulated proton diffusion kinetics.

Another strategy to lower proton and water activities where it is mostly needed, i.e., at the electrode surface, is by forming a solid electrolyte interphase (SEI).^[^ ^45^ ^]^ An ideal SEI layer would directly prevent access of the aqueous electrolyte to the electrode surface while allowing the charge‐carrying cations through. However, a functioning SEI layer is lacking in aqueous batteries with dilute electrolytes, owing to both the narrow ESW of water and the high solubilities of SEI species. Such a challenge has been partly mitigated by the use of highly concentrated aqueous electrolytes (i.e., WiSEs)^[^ ^46^ ^]^ or electrolytes with a molecular crowding agent,^[^ ^47^ ^]^ in which the activity of water is sufficiently suppressed. The main SEI components found under such conditions are LiF and Li_2_CO_3_. Specifically, LiF is proposed to stem from the decomposition of TFSI^−^ anions, either electrochemically^[^ ^46^ ^]^ or chemically,^[^ ^40^ ^]^ while Li_2_CO_3_ is observed in aqueous electrolytes with dissolved CO_2_ molecules.^[^ ^48^, ^49^ ^]^ Nevertheless, when further lowering the electrolyte salt concentration, both species may be attacked by protons in the electrolyte or de‐inserted from the electrode material and subsequently dissolve in the electrolyte (LiF + H^+^ → Li^+^ + HF, ${\mathrm{CO}}_{3}^{2-}+2H^{+}\to CO_{2}+H_{2}O$).

Other chemical species found at the electrode‐electrolyte interphase may also be sensitive to the proton activity in the electrolyte. For example, zinc hydroxide sulfate (Zn_4_(OH)_6_SO_4_ · *x*H_2_O, ZHS) is often observed as a side‐product in aqueous Zn‐ion batteries,^[^ ^50^ ^]^ whose formation is attributed to the precipitation of insoluble sulfate salts when the local electrolyte pH increases (proton insertion/consumption). Such a process is largely reversible, as ZHS will be re‐dissolved in the electrolyte by the de‐inserted proton. Therefore, ZHS can be seen as a pH‐buffering species for aqueous Zn‐ion batteries.

**Table 1:** Representative proton‐involving (electro)chemical reactions in aqueous batteries.

Category	Representative reaction	Ref.
Bulk and surface proton storage	MnO_2_ + *xe* ^−^ + *x*H^+^⇌H_*x*_MnO_2_	[14, 22]
	RuO_*x*_(OH)_*z*_ + δ*e* ^−^ + δH^+^⇌RuO_*x* − δ_(OH)_*z* + δ_	[26]
	**─**C═O + *e* ^–^ + H^+^ ⇌ ─C─O─H	[30]
Spontaneous ionization/dissociation	2H_2_O⇌H_3_O^+^ + OH^−^	–
	HA⇌H^+^ + A^−^	–
	$M{\left(H_{2}O\right)}_{6}^{2+}⇌{\left[M{\left(H_{2}O\right)}_{5}\mathrm{OH}\right]}^{+}+H^{+}$	[37, 38]
	$5V_{2}O_{5}+3H_{2}O\to HV_{10}O_{28}^{5-}+5H^{+}$	[35]
Electrochemical decomposition	2H^+^ + 2*e* ^−^ → H_2_	–
	2H_2_O → 4*e* ^−^ + 4H^+^ + O_2_	–
	C + 2H_2_O → 4*e* ^−^ + 4H^+^ + CO_2_	[35]
	2Al + 6H^+^ → 2Al^3 +^ + 3H_2_	–
Self‐discharge/dissolution	2Li_*x*_(Host) + 2*x*H_2_O → 2Host + 2*x*LiOH + H_2_	[42]
	LiF + H^+^ → Li^+^ + HF	[35]
	Li_2_CO_3_ + 2H^+^ → 2Li^+^ + H_2_O + CO_2_	[35]
	$Zn_{4}{\left(\mathrm{OH}\right)}_{6}SO_{4}\cdot xH_{2}O+6H^{+}\to 4Zn^{2+}+{\mathrm{SO}}_{4}^{2-}+\left(6+x\right)H_{2}O$	[50]
	MnO_2_ + 2*e* ^−^ + 4H^+^⇌Mn^2 +^ + 2H_2_O	[51, 52]

## 4 How do We Better Understand, Control, and Utilize Proton Activities in Aqueous Batteries?

Understanding the energetics and the dynamics of protons is key to realizing functioning aqueous rechargeable batteries. First, when the protons are foreseen for charge storage, determining the de‐/insertion mechanism is paramount. For instance, the intercalation of hydronium rather than protons into active material is curious. Possibly, the high dehydration energy of H_3_O^+^ prevents the intercalation of dehydrated H^+^, even in highly acidic electrolytes, although the larger size of H_3_O^+^ should be sterically prohibited. In order to clarify the situation, the concentration of protons in the electrolyte should be controlled as it has the most dominant impact on proton activity. A tremendous amount of pH regulation strategies have been developed to mitigate the detrimental effect of HER and other parasitic side reactions, such as novel electrolyte formulation, electrolyte decoupling, and artificial passivation layer formation.^[^ ^53^ ^]^ In the case of aqueous Zn‐ion batteries, since metallic zinc has an electrode potential much lower than that of the standard hydrogen electrode (−0.76 V), HER is thermodynamically favorable, posing a fundamental change to the successful implementation of the new chemistry. In this regard, highly polar dimethylacetamide and trimethyl phosphate molecules have been shown to increase the electron density of water protons, hence suppressing the water activity and the associated HER.^[^ ^54^ ^]^ Alternatively, Gao et al. reported that heavy water (D_2_O)‐based electrolytes exhibit a wider ESW than that of normal water (H_2_O), showing limited HER/OER activity, less pH value change, and reduced side product formation.^[^ ^55^ ^]^ Moreover, the introduction of a pH buffer in the electrolyte is promising to stabilize the redox chemistry for analysis, or even to enhance performance. For instance, the addition of buffer substances to the electrolyte has improved the reversibility of the Mn^2+^/MnO_2_ deposition/dissolution mechanism in Zn/MnO_2_ batteries.^[^ ^56^ ^]^ The same concept can be applied to other cell chemistries, e.g. aqueous Li‐ion, in which HER and/or active material dissolution is to be mitigated.

Even if the concentration is maintained, proton activity is also strongly influenced by the environment. The presence of other ions in the electrolyte may shield electrostatic interactions between protons and their surroundings. As the ionic strength increases, protons experience less interaction with other charged species, reducing their activity despite a constant concentration. The chemistry and structure of the electrode surface can influence proton transport, affecting local proton activity. The electrochemical reactions of protons on the surface/sub‐surface of the electrode materials experience a different environment than protons in the bulk electrolyte. To mitigate these challenges, the ionic strength of the electrolyte can be carefully tuned by adding inert supporting electrolytes (e.g., Na_2_SO_4_, KNO_3_),^[^ ^57^ ^]^ which helps stabilize the activity coefficient of protons, making their activity less sensitive to changes from the environment. Alternatively, electrode surface modification, such as grafting a functionalized coating layer and doping the material with anticatalytic elements, may also be a viable solution to selectively interact with protons.^[^ ^58^ ^]^ Furthermore, the coupling between proton reaction and diffusion needs to be investigated to understand the underlying mechanism. The two processes collectively determine the overall behavior of the proton activity. For example, the proton reaction often depends on the local availability of protons, which is determined by their diffusion kinetics. This may also result in a drastic difference in the proton concentration near the electrode surface and in the bulk solution, affecting the performance of the electrode. In this regard, AIMD simulations and machine learning accelerated MD simulations will be indispensable tools, which can provide complementary information on the structure, dynamics, and reactivity of bulk, interface, and interphase with quantum mechanical accuracy.^[^ ^44^, ^59^ ^]^ They will also be crucial for understanding the electrostatic aspect of proton activities, either due to the electric field from the double layer^[^ ^60^ ^]^ or counter‐ions at concentrations beyond infinitely dilute conditions.^[^ ^61^ ^]^ A key to this endeavor from the molecular modeling community is to establish model systems and to devise physical quantities that are accessible in experiments. This will not only increase the credibility of simulation methods but also maximize their powers in providing new physical insights and non‐trivial predictions.

Protons clearly participate in side reactions of which dissolution of the active materials pertains to one of the most challenging issues. It has already been observed that metal dissolution is strongly impeded in electrolytes of high ionic strength, a consequence that has been exploited to realize reversible cycling of novel active materials otherwise highly prone to dissolution. For instance, Dubouis et al. successfully demonstrated reversible lithium intercalation in layered transition metal halides using a highly concentrated electrolyte (5 m of LiFSI in dimethyl carbonate), which cannot be cycled in dilute electrolytes owing to extensive vanadium dissolution.^[^ ^62^ ^]^ The same concept is promisingly extendable to aqueous electrolytes as only a fraction of available ion‐host metal oxides remain insoluble under dilute salt concentrations of aqueous electrolytes.

Alternatively, high proton activities may enable new reaction mechanisms, corroborated by the recently proposed electrolytic Zn─Mn chemistry (MnO_2_ + 2*e* ^−^ + 4H^+^⇌Mn^2 +^ + 2H_2_O).^[^ ^51^, ^52^ ^]^ Such a process enables a two‐electron transfer of manganese, offering a doubled theoretical capacity (616 mAh g^−1^) and a 50% increase in redox potential (1.99 V vs Zn). While plenty of details remain to be optimized (e.g., electrolyte formulation, deposition kinetics, Zn corrosion mitigation, etc.), it is evident that the proton activity in the electrolyte plays a pivotal role in altering the charge storage mechanism of the aqueous chemistry.^[^ ^63^ ^]^

Regardless of electrolyte composition, the presence of an aqueous electrolyte in a battery cell operating beyond 1.23 V will inevitably trigger water decomposition. The only strategy to maintain a high‐voltage aqueous battery with a practically relevant lifetime is thus to separate the electrode from the electrolyte with an interphase, at both electrodes. The need to suppress HER at the negative electrode is obvious. However, an effective solid electrolyte interphase remains to be realized. Furthermore, the formation of a high‐performance cathode‐electrolyte interphase (e.g., CEI) on the positive electrode could prove to be more critical for aqueous than non‐aqueous cell chemistries. Although O_2_ evolution is sluggish, oxidation of the conductive carbon additives occurs more readily and will eventually deteriorate the electronic conductivity.^[^ ^64^ ^]^ Since conductive carbon is an integral part of an electrode coating, we call for more attention on how to stabilize it and assess the impact of side reactions at the carbon surface.

Finally, considering that several of the processes discussed above occur inside the operating cell beyond equilibrium conditions, a fundamental understanding of the governing mechanisms underpinning battery performance relies on *operando* analyses,^[^ ^65^ ^]^ preferably with a non‐destructive nature.^[^ ^66^, ^67^ ^]^ Tremendous progress is being made in the development and application of analytical techniques for batteries, which will be essential to mitigate and master proton activities in aqueous rechargeable batteries.

## Conflict Of Interest

The authors declare no conflict of interest.
